# The association of socioeconomic status on kidney transplant access and outcomes: a nationwide cohort study in Taiwan

**DOI:** 10.1007/s40620-024-01928-5

**Published:** 2024-04-18

**Authors:** Tung-Ling Chung, Nai-Ching Chen, Chun-Hao Yin, Ching-Chih Lee, Chien-Liang Chen

**Affiliations:** 1 Division of Nephrology, Kaohsiung Veterans General Hospital, Kaohsiung, Taiwan; 2 Departments of Neurology, Kaohsiung Chang Gung Memorial Hospital and Chang Gung University College of Medicine, Kaohsiung, Taiwan; 3 Department of Medical Education and Research, Kaohsiung Veterans General Hospital, Kaohsiung, Taiwan; 4 Division of Otolaryngology, Kaohsiung Veterans General Hospital, Kaohsiung, Taiwan; 5 National Yang Ming Chiao Tung University, Hsinchu, Taiwan; 6 Faculty of Medicine, National Sun Yat-sen University, No. 70 Lien-hai Road, Kaohsiung, 804201 Taiwan

**Keywords:** Socioeconomic status, Kidney transplant access, Kidney transplant outcome, Survival

## Abstract

**Background:**

Conflicting evidence exists regarding the relationship between socioeconomic status and access to or outcomes after kidney transplantation. This study analyzed the effects of individual and neighborhood socioeconomic status on kidney transplant access and outcomes in Taiwan.

**Methods:**

We used a retrospective cohort study design and performed comparisons using the Cox proportional hazards model after adjusting for risk factors. Data were collected from the National Health Insurance Bureau of Taiwan data (2003–2012).

**Results:**

Patients with high individual and neighborhood socioeconomic status had higher chances of receiving kidney transplants than those with low individual and neighborhood socioeconomic status [adjusted hazard ratio (aHR) = 2.04; 95% CI: (1.81–2.31), *p* < 0.001]. However, there were no significant differences in post-transplant graft failure or patient mortality in Taiwan between individuals of varying socioeconomic status after five years. When we stratified kidney transplants by domestic and overseas transplantation, there were no significant differences in post-transplant mortality and graft failure, but individuals who received a kidney graft in Taiwan with high individual and neighborhood socioeconomic status experienced lower risks of graft failure (aHR = 0.55; [95% CI 0.33–0.89], *p* = 0.017).

**Conclusion:**

A relevant disparity exists in accessing kidney transplantation in Taiwan, depending on individual and neighborhood socioeconomic status. However, results post transplantation were not different after five years. Improved access to waitlisting, education, and welfare support may reduce disparities.

**Graphical Abstract:**

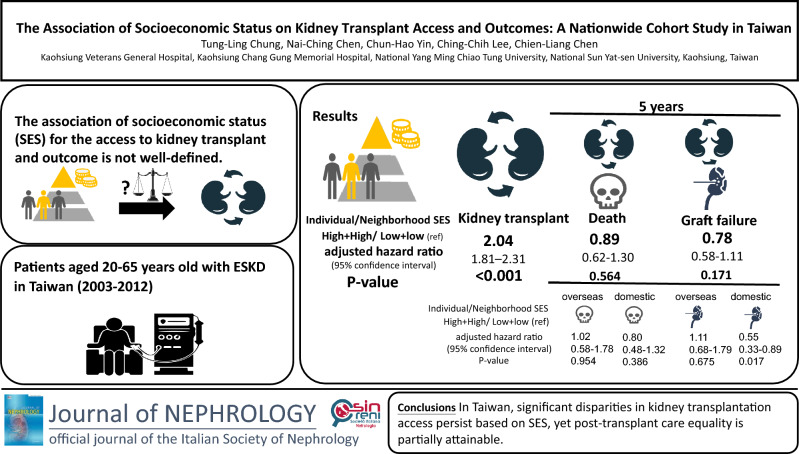

**Supplementary Information:**

The online version contains supplementary material available at 10.1007/s40620-024-01928-5.

## Introduction

Taiwan has the highest incidence of end-stage kidney disease (ESKD) worldwide. The prevalence of ESKD reached 3546 per million people by 2021 [1]. Furthermore, at least 20% of Taiwan’s population consists of elderly people, making it an extreme-aged society, according to the definition of  “aged society” as having more than 14% of elderly people [2]. Aging is associated with an increased prevalence of chronic diseases, such as hypertension and diabetes mellitus, and the use of herbs with and without aristolochic acid in Taiwan has contributed to the incidence of ESKD [3, 4]. Taiwan’s current healthcare system, the National Health Insurance (NHI), is a single-payer compulsory social insurance plan providing healthcare to all Taiwanese residents. Coverage reached 99% of the population at the end of 2004. The health insurance system is inexpensive and provides care to patients undergoing dialysis. At present, the 5-year survival rate for dialysis patients in Taiwan is nearly 60%, compared to 40.3% and 41.3% for US hemodialysis patients and peritoneal dialysis patients, respectively, but similar to Korean dialysis patients [1, 5]. Care for patients undergoing dialysis is a financial burden on the healthcare system. As they live longer, the number of dialysis patients has increased. The medical community prefers transplantation to dialysis because of its advantages on life expectancy, health outcomes, quality of life, and lower medical costs [6]. Kidney transplantation rates vary across countries. In 2021, the incidence of KT was highest in Brunei Darussalam (109 per million general population [PMP]), followed by the U.S. (77 PMP), Jalisco (58 PMP), and Israel (56 PMP). Most European countries reported rates of 30–40 PMP [1]. However, KT rates in Taiwan are lower, at only 15 PMP. Only approximately 345 people in Taiwan undergo successful kidney transplantation per year, accounting for 0.4% of ESKD patients, compared with 50% in England and Scandinavian countries [1].

Access to organ transplantation is determined by the availability of organs and healthcare. There are two organ sharing systems: the China Organ Registry Center, which was established in 2008, and the Taiwan Organ Registry and Sharing Center, which was established in 2002 to serve as a bridge between donors, recipients, organ procurement hospitals, and organ transplantation hospitals [7–10]. Only deceased donor and living-related kidney transplants are currently performed in China, and there have been no living-unrelated donors since January 1, 2015 [7–9]. In Taiwan, patients can register on kidney transplant waiting lists in Taiwan and China because of China’s political policy. Hence, they can receive kidney transplants in both China and Taiwan. However, when receiving kidney transplants in China, patients must pay the medical cost of transplantation, as the Taiwanese health insurance does not cover the medical fees in China. Fundamental inequalities exist in access to transplantation after waiting list acceptance for adults in both the USA and Europe [11–17]. Medical conditions and non-medical factors affect whether patients receive a transplant. Patient characteristics include age; chronic diseases, such as diabetes mellitus; gender [11–14]; race [16]; socioeconomic factors, such as educational level, and area of residence, a proxy of income [11, 12]. Few studies have examined the interactions between individual socioeconomic status and neighborhood deprivation, and so far the findings are heterogeneous [12–15, 17, 18]. Death rates for adults with low socioeconomic status were the highest in high-socioeconomic status neighborhoods and lowest in low-socioeconomic status neighborhoods [18]. The Taiwanese government reports that 95% of Taiwan's population is of Han Chinese ethnicity. Over 2% of the population consists of indigenous Taiwanese. Twenty-one thousand Westerners live in Taiwan, accounting for 0.1% of its total population. There were 300,000–400,000 South Asian residents (Indonesians, Filipinos, Thai, Vietnamese) in Taiwan from 2003 to 2012, representing 1.3–1.7% of the country's population [19]. Hence, choosing the Taiwanese population as the study group eliminated racial bias since the presence of socioeconomic disparities may impact long-term graft survival. Further research is needed to examine the mechanisms contributing to disparities in kidney transplantation and post-transplant survival, to ultimately intervene with culturally sensitive approaches.

We designed a population-based study using data from the Taiwan NHI Administration to investigate the role of individual and neighborhood socioeconomic status on access to domestic and overseas kidney transplantation. Furthermore, we investigated survival rates after transplantation according to different subgroups.

## Methods

### Ethics statement

This study was approved by the Ethics Committee of Kaohsiung Veterans General Hospital (VGHKS18-CT10-07). The requirement for informed consent was waived because the data were anonymized.

### Database

The NHI Program database contains registration files and original claims data for reimbursement. Large computerized databases derived from this system by the NHI Administration are provided to scientists in Taiwan for research purposes. This dataset can be accessed from the NHI Research Database (NHIRD) website (http://nhird.nhri.org.tw/). The NHIRD provides extensive information, including age, sex, date of admission, mortality, International Classification of Diseases, Ninth Revision, Clinical Modification (ICD-9-CM) medical procedures, diagnostic codes, comorbidities, and emergency care details. In Taiwan, all chronic dialysis payments are covered by the NHI program; in other words, all patients with ESKD (based on ICD-9-CM code 585) are included in the NHIRD. The diagnosis of ESKD with ICD-9-CM code 585 can only be made by certified nephrologists and revalidated by nephrologists selected from other hospitals. In the original study, the NHI Bureau of Taiwan randomly reviewed the charts of 1 out of every 100 ambulatory cases in the year 2000, and the study groups were followed up from 2003 to 2012 based on Taiwan’s NHIRD. For the protection of personal information, all data were de-identified as secondary data.

## Measurement

### Patient selection and definition

This study included all working-age patients (20–65 years old) with ESKD in Taiwan between 2003 and 2012 using the database of major illnesses (based on the ICD-9-CM codes 585.6), with patient demographics obtained at diagnosis of ESKD, including age, sex, medical comorbidities, number of admissions, and hospital characteristics. We obtained data on all patients who had received kidney transplants based on the ICD-9-CM codes V42.0. In Taiwan, all patients undergoing kidney transplantation receive a medical claim review by nephrologists or urologists from different medical facilities. To identify domestic or overseas kidney transplants, we checked transplant recipients (ICD-9-CM codes V42.0) to determine whether they had undergone kidney transplant surgery (ICD-9-CM codes 76020A, 76020B, and 97416K). Kidney transplantation and surgery codes were defined as domestic kidney transplants. A kidney transplant code without a surgery code was defined as an overseas kidney transplant. Overseas kidney transplant recipients were validated using the NHI-based registry of catastrophic illness to exempt co-payment. Allograft failure was identified based on the patient’s dialysis re-entry (defined as > 10 dialysis sessions 90 days after transplantation), including hemodialysis and peritoneal dialysis (ICD-9-CM codes 58001C, 58027C, 58029C, and 58002C). Patient death could be identified by the NHI system. Non-transplant patients were censored at the date of death or end of the follow-up period. Living patients were censored at the end of the follow-up period. Kidney transplantation was considered as a time-dependent covariate.

We selected individual and neighborhood socioeconomic status and survival as the main independent variables.

### Individual-level measures

In this study, we used income-related insurance payment amounts as a proxy for individual socioeconomic status. After confirming that the use of this proxy was validated in a previous study [20], we selected US$698 (New Taiwan Dollar [NT$] 21,500) per month as the low-income cut-off point because this was the government-stipulated minimum wage for full-time employees in Taiwan in 2007 [21].

### Neighborhood-level socioeconomic status

We divided Taiwan into 369 areas, including townships and small cities, for socioeconomic analysis. To characterize township-level socioeconomic conditions, we first identified information from Taiwan’s census statistics depicting neighborhood and household economic conditions. Variables associated with known socioeconomic differences were also included. We defined neighborhood socioeconomic status based on the percentage of households and average family income in Taiwan. In this census, the neighborhood household income of a township was the per-capita income determined by the Taiwan Ministry of Finance based on 2003 tax statistics (Supplementary Figure S1) [21]. The neighborhoods were sorted according to their median incomes; high- and low-socioeconomic status neighborhoods had higher and lower-than-median household incomes, respectively. We stratified population density, number of outpatients followed-up, number of inpatients, and residential urbanization [20, 22].

### Others

In this study, other comorbidities were classified as congestive heart failure (ICD-9-CM code 428.x), chronic obstructive pulmonary disease (ICD-9-CM codes 491.2x, 493.2x, and 496), hypertension (ICD-9-CM code 401.9), diabetes mellitus (ICD-9-CM code 250.x), stroke (ICD-9-CM code 433.xx–434.xx), and coronary artery disease (ICD-9-CM codes 410.x–414.x). We categorized diseases with ≥ 3 outpatient visits into the high outpatient group and those with ≥ 1 inpatient admission into the high inpatient group. We used the accreditation level to distinguish hospitals as medical centers, regional hospitals, or district hospitals.

### Statistics

Statistical analyses were performed using the Statistical Product and Service Solutions (SPSS) software (version 22; SPSS, Inc., Chicago, IL, USA) and Statistical Analysis Software (SAS) version 9.3. Pearson’s chi-squared test was used to analyze categorical variables (level of urbanization, sex, category, and geographic region of residence) and hospital characteristics (medical center, district, and regional). The primary outcome was the association between individual and neighborhood socioeconomic status and access to kidney transplants, including deceased-donor or living-donor kidney transplants. The secondary outcome was the association between individual and neighborhood socioeconomic status and recipient mortality or graft failure, which was analyzed using the Kaplan–Meier survival curve and log-rank test. The Cox proportional hazards regression model was used to compare the results of different socioeconomic status categories before and after adjusting for patient characteristics (sex, age, and area of residence), comorbidities, and hospital characteristics (medical center, district, and regional). Statistical significance was defined as a two-sided *p*-value of < 0.05.

## Results

### Study process flowchart (Fig. 1)

**Fig. 1:**
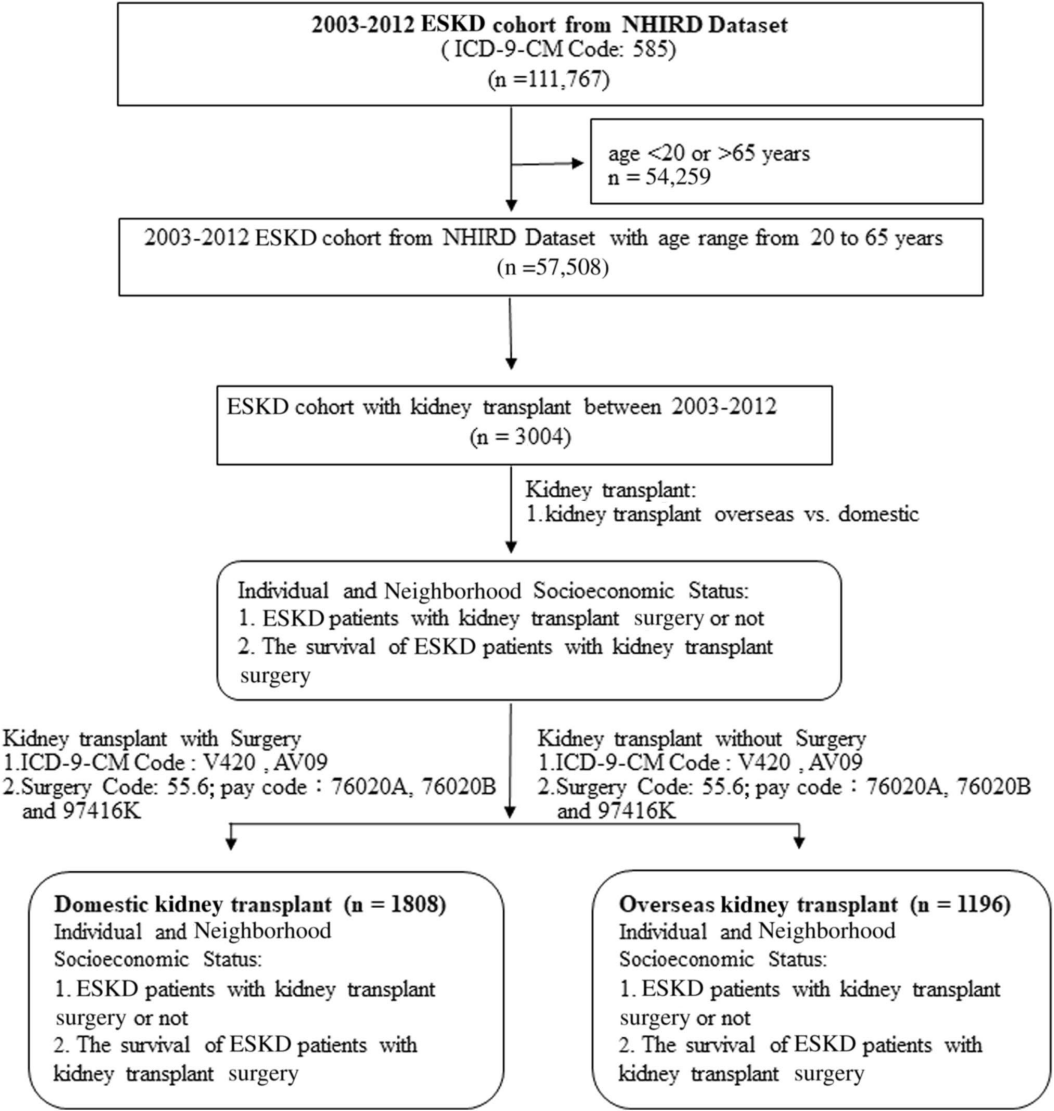
Study process flowchart

Overall, 57,508 patients with ESKD aged 20–65 years were included in this study. Only 3004 patients (2.7%) received kidney transplants.

### Demographic data and socioeconomic status characteristics

Table 1 shows the distribution of demographic and social variables, and how the variables differed depending on socioeconomic status for all patients. Compared with the disadvantaged socioeconomic status group, the advantaged socioeconomic status group was younger, consisted of more men, had higher turnaround times for receiving kidney transplants, and experienced less congestive heart disease, diabetes mellitus, and cardiovascular disease (*p* < 0.01).

**Table 1:** Baseline characteristics of ESKD patients within working age (20–65 years) between 2003 and 2012, *n* = 57,508

Variables	High SES	Low SES	*p* value
	*N* = 27,866 (%)	*N* = 29,642 (%)	
Age, years (mean ± SD)	51 ± 9	52 ± 10	< 0.001
*Sex*			< 0.001
Male	15,443 (55%)	15,310 (51%)	
Female	12,423 (45%)	14,332 (48%)	
*Number of in-patient hospital admissions before 1 year*			< 0.001
High (> 3)	3268 (12%)	4828 (14%)	
Low (0–3)	24,598 (88%)	25,360 (86%)	
*Hospital characteristics*			< 0.001
Medical center	11,190 (40%)	11,471 (39%)	
Regional	9398 (34%)	10,121 (34%)	
Other	7278 (26%)	8050 (27%)	
*Neighborhood SES*			< 0.001
Advantaged	5058 (18%)	6470 (22%)	
Disadvantaged	22,808 (82%)	23,172 (78%)	
*Region*			< 0.001
North	16,473 (60%)	19,454 (65%)	
South	11,393 (40%)	10,188 (35%)	
*Comorbidity*			
*CHF*			< 0.001
Yes	3751 (14%)	5009 (17%)	
No	24,115 (86%)	24,633 (83%)	
*COPD*			0.003
Yes	1322 (5%)	1566 (5%)	
No	26,544 (95%)	28,076 (95%)	
*PVD*			< 0.001
Yes	383 (1%)	515 (2%)	
No	27,483 (99%)	29,127 (98%)	
*DM*			< 0.001
Yes	9544 (34%)	11,237 (38%)	
No	18,322 (66%)	18,405 (62%)	
*CVD*			< 0.001
Yes	2065 (7%)	3016 (10%)	
No	25,801 (83%)	26,626 (90%)	
*Kidney transplant*			< 0.001
Yes	1764 (6%)	1094 (4%)	
No	26,102 (94%)	28,548 (96%)	

*SES* socioeconomic status, *SD* standard deviation, *CHF* congestive heart failure, *COPD* chronic obstructive pulmonary disease, *PVD* peripheral vascular disease, *CVD* cerebrovascular disease, *DM* diabetes mellitus

### The association of individual and neighborhood socioeconomic status on access to kidney transplantation (Fig. 2 and Table 2)

**Fig. 2:**
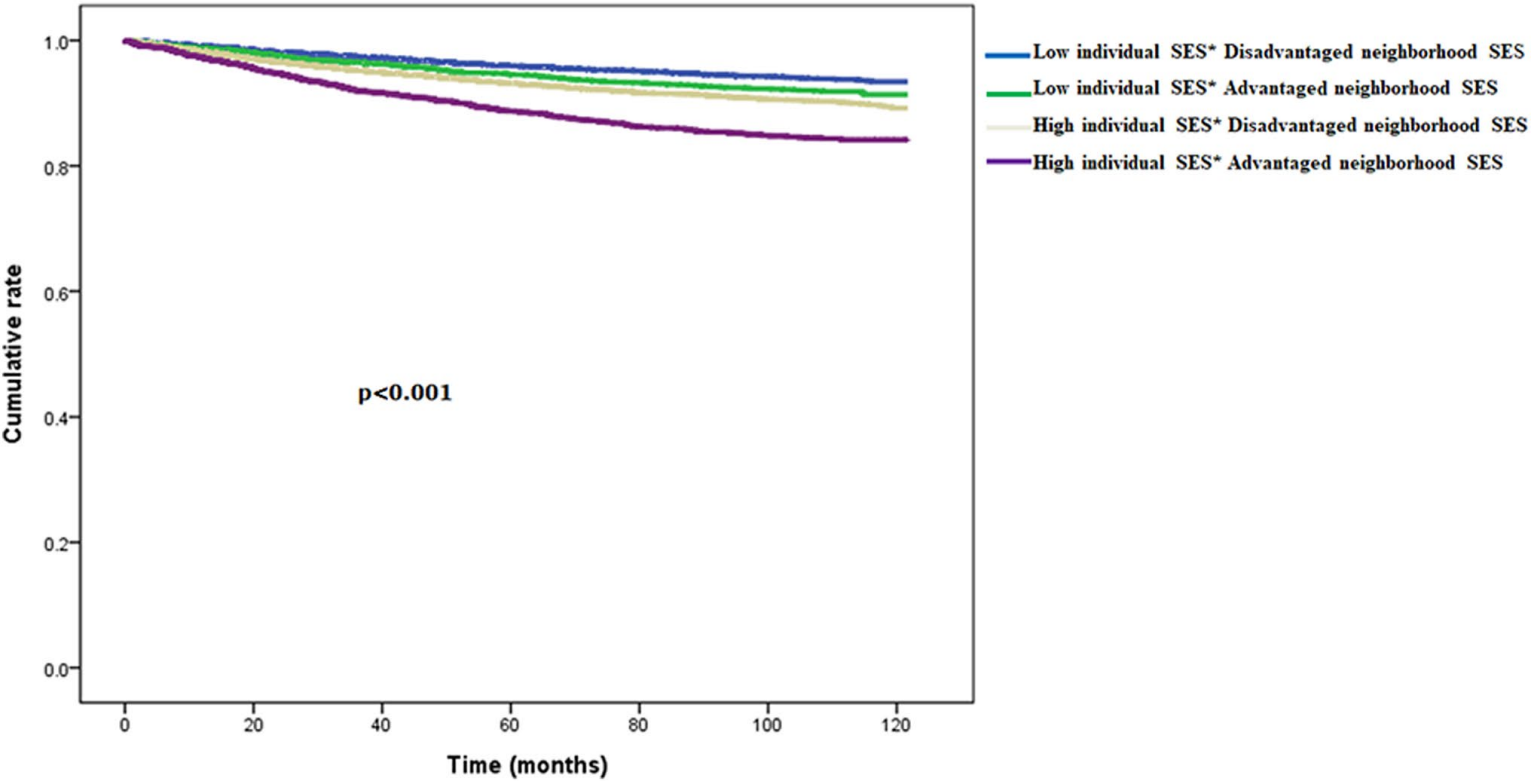
The association of individual and neighborhood socioeconomic status on access to kidney transplantation (the event of interest is kidney transplant)

**Table 2:** Multivariate Cox regression analysis for ESKD patients receiving kidney transplants

Variables	aHR (95% CI)	*p* value
Age	0.95 (0.95–0.95)	< 0.001
Gender—male	1.11 (1.08–1.20)	0.005
Individual SES and neighborhood SES		
Low individual SES in disadvantaged neighborhood	1	
Low individual SES in advantaged neighborhood	1.32 (1.16–1.53)	< 0.001
High individual SES in disadvantaged neighborhood	1.65 (1.51–1.81)	< 0.001
High individual SES in advantaged neighborhood	2.04 (1.81–2.31)	< 0.001
Hospital characteristics—medical center	1.72 (1.61–1.85)	< 0.001
Region-North	1.09 (1–1.19)	0.049
*Comorbidity*		
CHF—yes	0.63 (0.53–0.75)	< 0.001
DM—yes	0.52 (0.46–0.58)	< 0.001
CVD—yes	0.79 (0.63–0.97)	0.028

*aHR* adjusted hazard ratio, *SES* socioeconomic status, *CHF* congestive heart failure, *COPD* chronic obstructive pulmonary disease, *PVD* peripheral vascular disease, *DM* diabetes mellitus, *CVD* cerebrovascular disease

Age, sex, and comorbidities can decrease access to kidney transplantation. The proportional hazard assumption tests were checked by the survival time as the horizontal axis and the logarithmic survival (Supplementary Figure S2) and Schoenfeld residual analysis (Supplementary Figure S3). We found that patients with higher individual socioeconomic status who lived in more advantaged neighborhoods had a higher adjusted hazard ratio [(aHR) = 2.04, 95% CI 1.81–2.31, *p* < 0.001] for kidney transplantation after adjusting for other variables than the patients with low individual socioeconomic status who lived in disadvantaged areas (Table 2). Moreover, patients with low individual socioeconomic status living in advantaged neighborhoods had a higher rate of kidney transplantation (aHR = 1.32, 95% CI (1.16–1.53), *p* < 0.001) even after adjusting for age, sex, and individual comorbidities (Table 2).

### The association of individual and neighborhood socioeconomic status on access to overseas or domestic kidney transplantation

Of those who received transplants, 39.8% of patients did so overseas and 60.2 percent underwent tranplantation in Taiwan. As shown in Table 3, for overseas kidney transplantation, patients with high individual socioeconomic status also had a higher transplantation rate than those with low individual socioeconomic status after adjusting for variables (aHR = 2.51, 95% CI 2.09–3.02, *p* < 0.001). For domestic kidney transplantation, patients with high individual socioeconomic status living in advantaged areas had the highest rate of kidney transplantation (aHR = 2.01, 95% CI 1.74–2.34, *p* < 0.001).

**Table 3:** Multivariate Cox regression analysis for ESKD patients to receive kidney grafts (including domestic and overseas)

Variables	aHR (95% CI)	*p* value
*Domestic*		
Individual SES and neighborhood SES		
Low individual SES in disadvantaged neighborhood	1	
Low individual SES in advantaged neighborhood	1.66 (0.89–1.26)	0.474
High individual SES in disadvantaged neighborhood	1.48 (1.33–1.65)	< 0.001
High individual SES in advantaged neighborhood	2.01 (1.74–2.34)	< 0.001
*Overseas*		
Individual SES and neighborhood SES		
Low individual SES in disadvantaged neighborhood	1	
Low individual SES in advantaged neighborhood	1.59 (1.32–1.93)	< 0.001
High individual SES in disadvantaged neighborhood	1.98 (1.72–2.28)	< 0.001
High individual SES in advantaged neighborhood	2.51 (2.09–3.02)	< 0.001

*aHR* adjusted hazard ratio, *SES* socioeconomic status; adjusted variables: *COPD* chronic obstructive pulmonary disease, *HTN* hypertension, *DM* diabetes mellitus, *CAD* coronary artery disease, *CKD* chronic kidney disease, *HR* hazard ratio, *CI* confidence interval

### The association of individual and neighborhood socioeconomic status on graft failure or patient mortality in overall kidney transplantation

In overall kidney transplantation of Taiwanese patients (domestic and overseas), we found no significant differences in 5-year graft failure or life mortality in groups sorted for individual or neighborhood socioeconomic status after adjusting for age, sex, outpatient follow-up duration, number of admissions, hospital characteristics, area of residence, and comorbidities (Table 4).

**Table 4:** Multivariate Cox regression analysis for 5-year survival among all kidney transplant patients

Variables	aHR (95% CI)	*p* value
*Graft failure or patient mortality*		
Individual SES and neighborhood SES		
Low individual SES in disadvantaged neighborhood	1	
Low individual SES in advantaged neighborhood	1.03 (0.83–1.27)	0.796
High individual SES in disadvantaged neighborhood	0.88 (0.68–1.13)	0.304
High individual SES in advantaged neighborhood	0.79 (0.62–2.2)	0.075
*Patient mortality*		
Low individual SES in disadvantaged neighborhood	1	
Low individual SES in advantaged neighborhood	0.97 (0.66–1.44)	0.911
High individual SES in disadvantaged neighborhood	0.81 (0.58–1.12)	0.206
High individual SES in advantaged neighborhood	0.89 (0.62–1.30)	0.564
*Graft failure*		
Low individual SES in disadvantaged neighborhood	1	
Low individual SES in advantaged neighborhood	0.99 (0.74–1.33)	0.961
High individual SES in disadvantaged neighborhood	1.05 (0.75–1.46)	0.779
High individual SES in advantaged neighborhood	0.78 (0.58–1.11)	0.171

*aHR* adjusted hazard ratio, *SES* socioeconomic status. Adjusted variables: *COPD* chronic obstructive pulmonary disease, *HTN* hypertension, *DM* diabetes mellitus, *CAD* coronary artery disease, *CKD* chronic kidney disease

### The association of individual and neighborhood socioeconomic status on graft failure and patient mortality in overseas and domestic kidney transplantation (Figs. 3, Fig. 4)

**Fig. 3:**
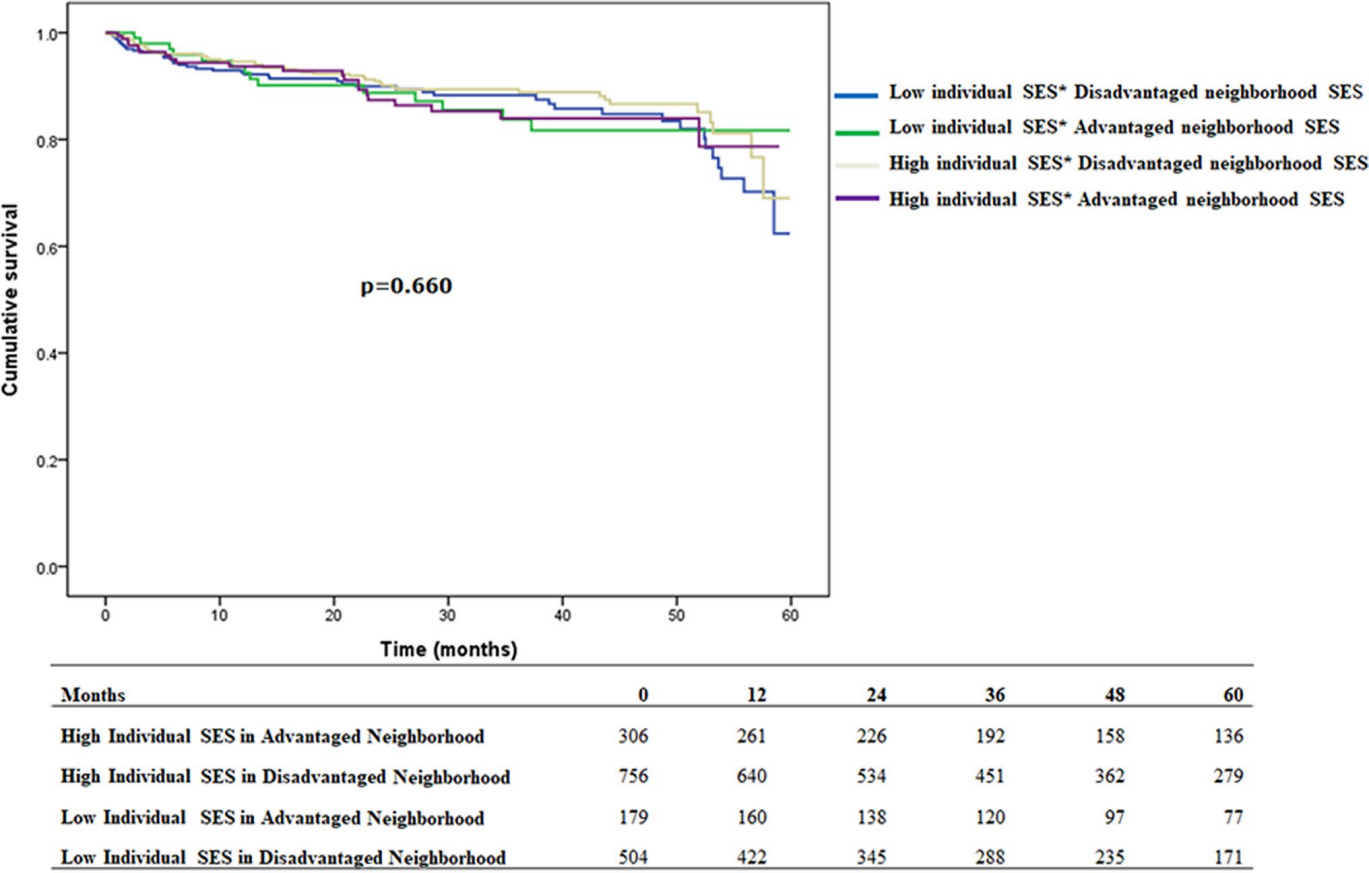
Individual and neighborhood socioeconomic status and patient mortality in domestic kidney transplantation

**Fig. 4:**
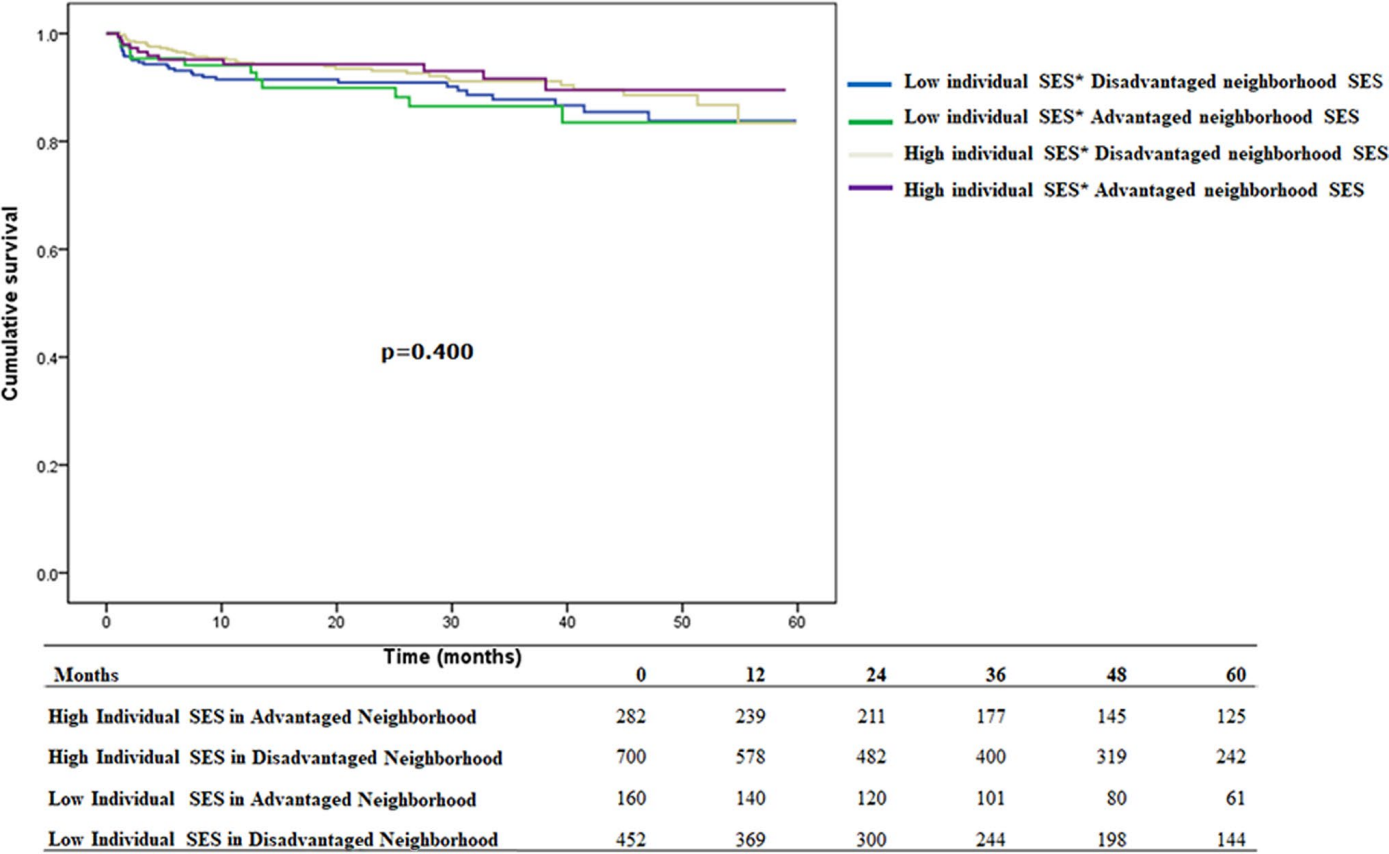
Individual and neighborhood socioeconomic status and graft failure in domestic kidney transplantation

In domestic kidney transplantation (Table 5), there were no differences in the survival of patients with different individual and neighborhood socioeconomic status (*p* > 0.05). However, for graft failure, the group with high individual socioeconomic status, living in advantaged areas had a lower risk (0.55; 95% CI, 0.33–0.89) compared to the group with low individual socioeconomic status living in disadvantaged areas. Regarding overseas kidney transplants (Table 5), we found no significant difference in mortality or graft failure between individual- and neighborhood-level socioeconomic status after adjusting for age, sex, outpatient follow-up duration, number of admissions, hospital characteristics, area of residence, and comorbidity (Table 5).

**Table 5:** Multivariate Cox regression analysis for 5-year survival among domestic/overseas kidney transplant patients

5-year survival among domestic kidney transplant patients		
Variables	aHR (95% CI)	*p* value
*Graft failure or patient mortality*		
Individual SES and neighborhood SES		
Low individual SES in disadvantaged neighborhood	1	
Low individual SES in advantaged neighborhood	0.89 (0.66–1.20)	0.444
High individual SES in disadvantaged neighborhood	0.96 (0.67–1.34)	0.805
High individual SES in advantaged neighborhood	0.60 (0.42–0.87)	0.006
*Patient mortality*		
Low individual SES in disadvantaged neighborhood	1	
Low individual SES in advantaged neighborhood	0.95 (0.55–1.64)	0.856
High individual SES in disadvantaged neighborhood	0.74 (0.47–1.17)	0.194
High individual SES in advantaged neighborhood	0.80 (0.48–1.32)	0.386
*Graft failure*		
Low individual SES in disadvantaged neighborhood	1	
Low individual SES in advantaged neighborhood	0.85 (0.57–1.27)	0.430
High individual SES in disadvantaged neighborhood	1.23 (0.79–1.91)	0.372
High individual SES in advantaged neighborhood	0.55 (0.33–0.89)	0.017

5-year survival among overseas kidney transplant patients		
Variables	aHR (95% CI)	*p* value
*Graft failure or patient mortality*		
Individual SES and neighborhood SES		
Low individual SES in disadvantaged neighborhood	1	
Low individual SES in advantaged neighborhood	1.05 (0.77–1.43)	0.775
High individual SES in disadvantaged neighborhood	0.73 (0.50–1.04)	0.084
High individual SES in advantaged neighborhood	1.02 (0.71–1.46)	0.911
*Mortality*		
Low individual SES in disadvantaged neighborhood	1	
Low individual SES in advantaged neighborhood	1.03 (0.59–1.80)	0.923
High individual SES in disadvantaged neighborhood	0.91 (0.56–1.46)	0.690
High individual SES in advantaged neighborhood	1.02 (0.58–1.78)	0.954
*Graft failure*		
Low individual SES in disadvantaged neighborhood	1	
Low individual SES in advantaged neighborhood	0.98 (0.64–1.52)	0.957
High individual SES in disadvantaged neighborhood	0.74 (0.45–1.21)	0.223
High individual SES in advantaged neighborhood	1.11 (0.68–1.79)	0.675

*aHR* adjusted hazard ratio, *SES* socioeconomic status. Adjusted variables: *COPD* chronic obstructive pulmonary disease, *HTN* hypertension, *DM* diabetes mellitus, *CAD* coronary artery disease, *CKD* chronic kidney disease, *HR* hazard ratio, *CI* confidence interval

## Discussion

This population-based study in Taiwan assessed the combined effects of individual and neighborhood socioeconomic status on access to kidney transplantation in patients with ESKD. We investigated post-transplant patient readmission rates and graft survival using data provided by the NHI system. Data show that only 3004 patients (2.7% of ESKD patients) underwent kidney transplantation between 2003 and 2012 (10 years). Furthermore, patients with lower individual and neighborhood socioeconomic status had a lower chance of kidney transplantation despite Taiwan’s universal care and donor care system. We divided the transplant population into two groups, those who received a kidney transplant overseas and in Taiwan; the disparity in access to kidney transplants remained, and was associated with patients’ socioeconomic status and place of residence. However, these inequalities, including mortality and graft failure, were less relevant after transplantation. In the subgroup analysis, disparities existed only within domestic kidney transplant graft survival rates.

Concerning overseas kidney transplants, patients with low socioeconomic status cannot afford the fees for travel, surgery, and immunosuppressive drugs. Thus, it is reasonable to assume that disparities exist between overseas kidney transplant recipients and domestic ones. Previous studies have shown an association between individual or area socioeconomic status inequalities and access to kidney transplantation [12–15, 17, 18]. Socioeconomically disadvantaged patients may have more comorbidities and lower medication adherence rates. Potential barriers along the path to transplantation have been identified in the United States. Several studies show that transplantation rates are associated with socioeconomic and geographical factors, and vary significantly across different ages, races, and sex [11–18]. There are also some findings regarding culturally-related and local barriers to kidney transplantation among Asians and Pacific Islanders, particularly those residing in resource-deprived neighborhoods [23]. Moreover, a higher social adaptability index is associated with an increased likelihood of being wait-listed for kidney transplant [24]. In our study, we found that only 12.2% of ESKD patients were on the domestic kidney transplant waiting list. Despite Taiwan being a small island, patients with lower individual socioeconomic status and those living in deprived neighborhoods also have less access to transplants. This suggests the existence of several barriers. According to the Taiwan organ-sharing system, patients with ESKD need to return to the outpatient clinic regularly to maintain waitlisting. Patients who did not return beyond 6 months were excluded from the waiting list. Despite the fact that access to the organ share system is universal and free of charge in Taiwan, patients with lower income experience other difficulties, such as the need to work extra hours to support family and the financial burden of transportation to attend medical appointments. Therefore, if patients have low socioeconomic status or live in disadvantaged areas, they may be less likely to return for checkups. Furthermore, low socioeconomic status is associated with lower education in understanding their rights to use social welfare and medical systems. A Canadian study found that Canadians with lower-socioeconomic status used primary care more frequently, but when adjusted for their healthcare needs, they were less likely to receive specialty care [25].

Socioeconomic factors drive outcomes in many areas of healthcare, including access to primary and specialty healthcare, compliance with therapy, ability to afford medications, and outcomes after surgical procedures. This suggests that access to primary care may be a pathway through which income inequality affects mortality rates. Socioeconomic status-driven health inequalities are pronounced even in countries with universal healthcare. A recent study from Canada showed higher mortality among men with lower income, education, and occupational status for several causes of death [26]. In England, socioeconomic status disparities persisted and even widened after the establishment of the National Health Service [28]^.^ The provision of universal coverage was insufficient to offset broader economic and social changes and inequalities. Socioeconomic status differences may be exacerbated by policies that require co-payment for drugs such as intravenous immunoglobulins, or that limit coverage to only several months post-transplantation [12]. However, in our study, individual and neighborhood socioeconomic status are not significantly associated with graft and patient survival after kidney transplantation. This can be attributed to the medical healthcare provided by Taiwan’s NHI and the social welfare system which provides free health coverage to low-income people.

The number of overseas transplants increased rapidly, perhaps because of improved transplantation outcomes, increased brokering activity, and organ supply in China. The number of overseas transplantations in Taiwan increased after 2000, peaked in 2005, and decreased after 2007 [27–29]. Taiwanese people can still register in China’s organ-sharing system and undergo kidney transplant in China because Taiwan is considered a part of China. Subgroup analysis revealed no significant differences in the overseas post-transplant mortality and graft failure rates. The Taiwanese healthcare system provides the same post-transplant care for overseas and domestic kidney transplant recipients. However, subgroup analysis for domestic kidney transplants revealed that individuals with high personal and neighborhood socioeconomic status experienced lower risks of graft failure (aHR = 0.55; [95% CI 0.33–0.89], *p* = 0.017) in domestic kidney transplants, suggesting that there is still need to reduce inequalities. The kidney transplant screening process could be one of the causes of the differences in graft failure. In Taiwan, all kidney transplant recipients receive an insurance that covers complement-dependent lymphocytotoxic cross-match tests to avoid rejection. However, further tests, such as donor-specific antibody screening (Luminex solid-phase assay or C1q assay) and antibody removal treatments, are not covered by this health insurance, and some patients cannot afford these tests. It is desirable that these treatments will be covered by the health insurance.

The strength of this study is that it used a nationwide cohort of ESKD patients to try to avoid selection biases and obtain a sufficient sample size to detect differences among patients with kidney transplantation with different individual and neighborhood socioeconomic status. Choosing the Taiwanese population as a study group may reduce the element of racial differences because over 95% of Taiwan's population is Han Chinese [19]. Furthermore, the diagnosis of ESKD related to dialysis and kidney transplantation is accurate because all medical charts must be reviewed by other hospital experts to confirm the diagnosis and avoid misclassification. However, this study has several limitations. In this study, we did not have detailed patient data, such as the cause of kidney disease and history of sensitization of patients to human leukocyte antigens in the NHIRD. Second, kidney transplantation candidates are selected and those with poor physical or mental health are less likely to be waitlisted. Confounding biases, such as low socioeconomic status, are also often associated with poorer overall health. Hence, the effects of low individual socioeconomic status may be underestimated because this analysis only included patients who successfully completed the process of evaluation and wait-listing. Even though multiple confounding variables such as medical comorbidity (Table 1) were controlled or adjusted for, residual confounding variables may still exist.

In conclusion, this study demonstrates the independent and combined effects of individual and neighborhood socioeconomic status on access to kidney transplantation, but not with the 5-year post-transplantation survival rate. Patients with individual and neighborhood deprivation were less likely to undergo kidney transplantation than those with individual deprivation and neighborhood advantage. However, differences in post-transplantation care are reduced through the follow-up offered by the Taiwanese healthcare system. Improved access to waitlisting through outreach clinics, education, or welfare support may further reduce disparities.

## Supplementary Information

Below is the link to the electronic supplementary material.

Supplementary file1 (DOCX 1420 KB)

## Data Availability

The datasets used, analyzed, or both during the current study are available from the corresponding author upon reasonable request.
