# Supplementary material for: The association of socioeconomic status on kidney transplant access and outcomes: a nationwide cohort study in Taiwan
Source: J Nephrol. 2024 Apr 18;37(6):1563–75. doi: 10.1007/s40620-024-01928-5 (PMC11473664; doi:10.1007/s40620-024-01928-5)

Supplementary Material:

Figure S1. The neighborhood-level SES was based on the average family income in a particular area in Taiwan. To analyze this, we used

<https://kiang.github.io/salary/map> to generate the images shown below.


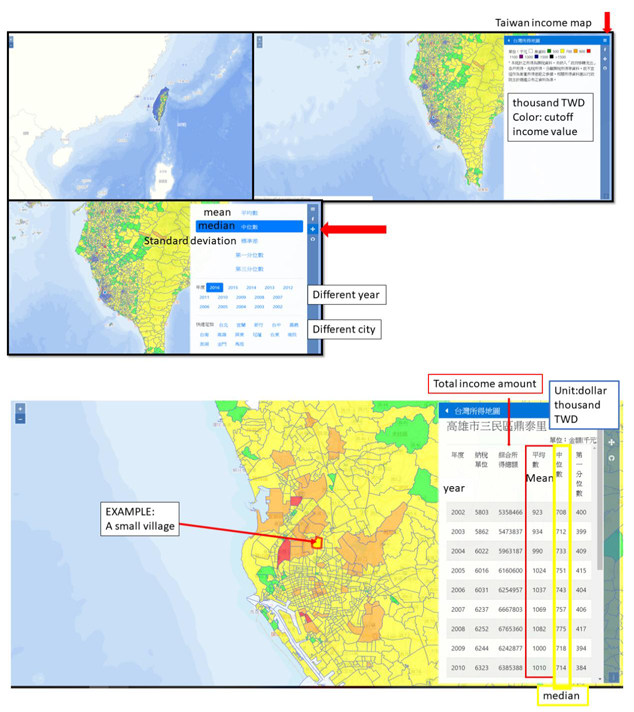


Figure S2. The log minus log plot of Kaplan-Meier curve to demonstrate the validity of the proportional hazards assumption under Cox regression was valid in the study. These four curves were roughly parallel.


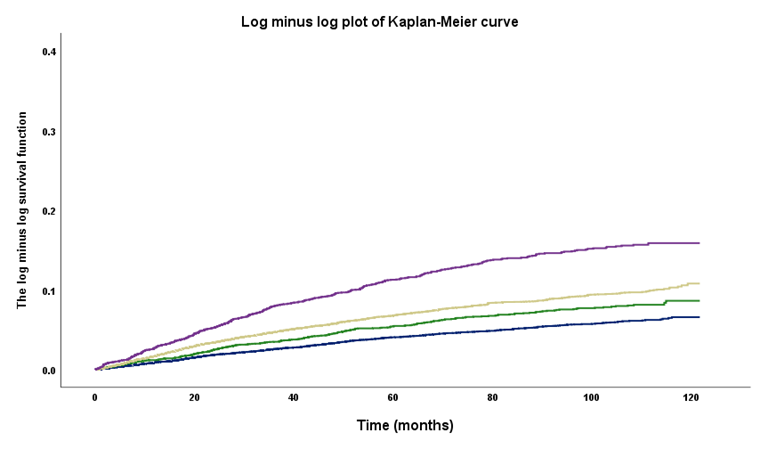


Low individual SES* Disadvantage neighborhood SES

Low individual SES* Advantage neighborhood SES

High individual SES* Disadvantage neighborhood SES

High individual SES* Advantage neighborhood SES

Figure S3. Schoenfeld residual was to check Cox proportional hazard model to make sure that there is no pattern with time.


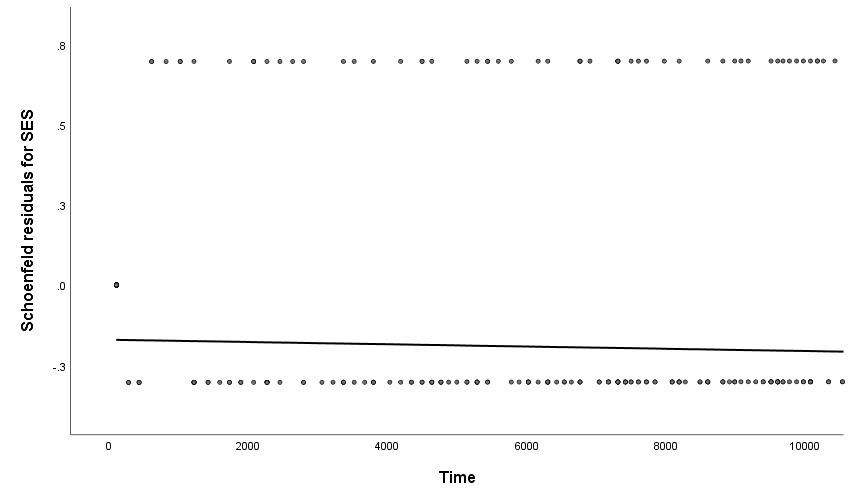

Supplement: Supplementary file 1 — Supplementary file1 (DOCX 1420 KB) [file 40620_2024_1928_MOESM1_ESM.docx]
